# Supplementary material for: Application of the Child Health and Nutrition Research Initiative (CHNRI) methodology to prioritize research to enable the implementation of Ending Cholera: A global roadmap to 2030
Source: PLoS One. 2022 May 26;17(5):e0264952. doi: 10.1371/journal.pone.0264952 (PMC9135262; doi:10.1371/journal.pone.0264952)
Supplement: S1 Table — (DOCX) [file pone.0264952.s001.docx]

# S4 Table. List of 93 research questions and their scores

| **Key priority** | **4D** | **Pillar** | **RQ** | **Weighted RPS** | **Weighted Rank** | **AEA** |
| --- | --- | --- | --- | --- | --- | --- |
| Yes | Delivery | OCV | What are the optimal oral cholera vaccine schedules (number of doses and dosing intervals) to enhance immune response and clinical effectiveness in children 1 to 5 years of age? | 88.8% | 1 | 81% |
| Yes | Delivery | OCV | What are potential delivery strategies to optimise oral cholera vaccine coverage in hard-to-reach populations (including during humanitarian emergencies and areas of insecurity)? | 87.4% | 2 | 76% |
| Yes | Delivery | Cross-cutting (OCV; WASH) | Is there additional benefit to adding WASH packages, for example household WASH kits, to an oral cholera vaccine campaign? | 87.1% | 3 | 77% |
| Yes | Delivery | OCV | What is the optimal number of doses of oral cholera vaccine to be used for follow up campaigns in communities previously vaccinated with a 2-dose schedule? | 86.9% | 4 | 76% |
| Yes | Delivery | OCV | Can the impact of oral cholera vaccine on disease transmission, morbidity and mortality be maximized by targeting specific populations and/or targeted delivery strategies? | 86.8% | 5 | 78% |
| Yes | Delivery | CM | What are the barriers and enablers for integrating cholera treatment into community case management by community health workers? | 86.8% | 6 | 75% |
| Yes | Delivery | WASH | What levels of coverage for relevant water, sanitation, and hygiene interventions is required in cholera hotspots to control and ultimately eliminate the risk of cholera? | 86.3% | 7 | 75% |
| Yes | Delivery | OCV | What impact does the timing of oral cholera vaccine use have on outbreak prevention and control? | 86.2% | 8 | 74% |
| Yes | Development | Epi / Sur / Lab | What is the impact of early diagnosis of cholera using a rapid diagnostic test at the point of care in a community setting compared to testing only in health facilities? | 86.1% | 9 | 75% |
| Yes | Delivery | OCV | How can the use of oral cholera vaccine in the controlled temperature chain (i.e. outside the cold chain) be leveraged to maximize the coverage or impact of vaccination in a field setting? | 85.9% | 10 | 75% |
| Yes | Delivery | Cross-cutting (All Pillars) | What is the incremental benefit of implementing a comprehensive interventions package (including water, sanitation, and hygiene, antibiotics, oral cholera vaccine, oral rehydration therapy) to reduce cholera mortality during an epidemic? | 85.7% | 11 | 74% |
| Yes | Delivery | OCV | What is the effectiveness and impact of different vaccination strategies for rapid response to cholera outbreaks (e.g., ring vaccination, case-area targeted interventions, etc)? | 85.3% | 12 | 74% |
| Yes | Development | Cross-cutting (OCV; WASH) | What is the most cost-effective package of water, sanitation, and hygiene and oral cholera vaccine in different situations, based on transmission dynamics in cholera hotspots? | 85.2% | 13 | 74% |
| Yes | Development | WASH | What are the most essential (or what is the minimum set of) infection, prevention, and control (IPC) interventions in cholera treatment facilities and oral rehydration points to reduce risk of transmission within these facilities? | 84.9% | 14 | 74% |
| Yes | Delivery | OCV | Are there immunisation strategies other than repeated mass campaigns that will be effective in preventing endemic or epidemic cholera? | 84.9% | 15 | 71% |
| Yes | Delivery | Cross-cutting (All Pillars) | What is the role and added value of CORTs (community outreach response teams) in enhancing case investigation and outbreak detection? | 84.6% | 16 | 71% |
| Yes | Development | OCV | Can oral cholera vaccine be co-administered safely and without interference with other vaccines during mass campaigns or during routine immunization visits (measles containing vaccines, yellow fever, typhoid, meningitis, pneumococcal conjugate vaccine)? | 84.3% | 17 | 72% |
| Yes | Delivery | Cross-cutting (WASH; CE) | What are effective strategies to scale up the use of household water treatment in controlling cholera outbreaks? | 84.1% | 18 | 71% |
| Yes | Development | Epi / Sur / Lab | How can we improve and fine-tune hotspot definition and identification at a district and sub-district level, such as micro-hotspots, including incorporating a population-based approach? | 84.1% | 19 | 72% |
| Yes | Development | WASH | Is improved access to safe water (e.g., water points and distribution networks) effective in controlling and preventing cholera outbreaks? | 84.0% | 20 | 74% |
| Q52 | Development | Epi / Sur / Lab | What are the optimal design(s) of surveillance systems (e.g., indicator-based, event-based, community-based, environmental, sentinel site surveillance) to monitor progress of the Cholera Roadmap? | 83.8% | 21 | 71% |
| Q101 | Delivery | Cross-cutting (WASH; CE) | What are the factors, practices, and behaviours that influence the uptake of different domestic hygiene or household interventions to support good hygiene and disinfection (or cleaning) practices for preventing and controlling cholera? | 83.5% | 22 | 70% |
| Q1 | Development | CM | What effect does treatment with antibiotics have on cholera transmission? | 83.3% | 23 | 71% |
| Int131 | Delivery | WASH | How can "design thinking" be used to improve the delivery / uptake of water, sanitation, and hygiene interventions? Design thinking focuses on understanding the needs of the people who will use the intervention and working with them to improve it. | 83.0% | 24 | 67% |
| Q63 | Description | OCV | What is the level and duration of herd protection from different vaccination schedules and strategies (e.g., one versus two doses, usefulness of annual single boosts to maintain herd protection, importance of vaccinating new-comers and former infants to maintain herd protection)? | 83.0% | 25 | 70% |
| Q53 | Development | Epi / Sur / Lab | What are the optimal surveillance tools (e.g., laboratory methods, case definitions, etc.) to monitor progress of the Cholera Roadmap? | 82.8% | 26 | 70% |
| Q103 | Delivery | Cross-cutting  (WASH; CE) | What is the effectiveness of different approaches and delivery mechanisms to improve the uptake of hygiene promotion activities? | 82.6% | 27 | 68% |
| Int3 | Delivery | Cross-cutting  (CM; CE) | What are the optimal strategies to empower mothers and / or women to adopt preventive strategies and seek appropriate care for cholera? | 82.6% | 28 | 66% |
| Q71 | Description | OCV | What are the factors influencing the duration of protection following use of oral cholera vaccine (includes target population and vaccination schedules)? | 82.6% | 29 | 67% |
| Q66 | Development | OCV | What are the relative costs and cost-effectiveness of different oral cholera vaccine delivery strategies? | 82.3% | 30 | 69% |
| Q120 | Development | Cross-cutting  (All Pillars) | What is the effectiveness of different communication platforms for behaviour change programmes and risk communication in cholera prevention and control programmes? | 82.3% | 31 | 67% |
| Q15 | Delivery | Cross-cutting  (CM; CE) | How, when, and where do people in the community seek care for cholera? What are the potential barriers (e.g. cultural and religious) to seeking timely cholera care. | 82.1% | 32 | 70% |
| Q51 | Delivery | Cross-cutting  (All Pillars) | What is the required coverage and use level for each of the cholera interventions (including water, sanitation, and hygiene, oral cholera vaccine, case management, and community engagement) needed to reduce transmission? | 81.7% | 33 | 68% |
| Q154 | Delivery | Cross-cutting  (CM; CE) | What are the solutions to overcoming the barriers (e.g., cultural and religious) to seeking timely cholera care? | 81.7% | 34 | 65% |
| Sur38 | Delivery | Cross-cutting  (All Pillars) | What are potential strategies to optimise cholera prevention and control interventions in hard-to-reach populations including during humanitarian emergencies and areas of insecurity. (Examples can include: innovative contingency vaccination strategies, use of community-based teams to maintain frontline worker safety, engaging law enforcement agencies and military, etc )? | 81.4% | 35 | 66% |
| Q57 | Description | Epi / Sur / Lab | How can combined epidemiological and genomic analysis of V. cholerae be used to better understand transmission dynamics and inform epidemiological models? | 81.2% | 36 | 68% |
| Int122 | Delivery | Cross-cutting  (OCV; CE) | What are prevailing knowledge, attitude, and practices in communities with respect to cholera vaccination? | 80.9% | 37 | 69% |
| Q108 | Development | Cross-cutting  (All Pillars) | What is the impact and cost-effectiveness of the different interventions in case-area targeted interventions (CATI) and how can the impact be optimised? | 80.9% | 38 | 65% |
| Q153 | Discovery | Epi / Sur / Lab | Research and development of novel and innovative diagnostic tests to accelerate the achievement of the Cholera Roadmap goals. | 80.8% | 39 | 66% |
| Q138 | Development | Epi / Sur / Lab | What are the optimal methods / strategies for environmental surveillance for V. cholerae? | 80.7% | 40 | 66% |
| Q133 | Development | OCV | What are the benefits and risks of vaccinating vulnerable populations in a cholera outbreak (for example: pregnant women, the elderly, those with severe acute malnutrition)? | 80.6% | 41 | 69% |
| Q3 | Development | CM | What is the optimal treatment schedule for antibiotic prophylaxis given to household contacts of cholera patients and does this have an effect on the magnitude, transmission and secondary attack rate of cholera outbreaks? | 80.5% | 42 | 70% |
| Q30 | Development | OCV | What are the long-term impacts of oral cholera vaccine on disease severity and mortality from subsequent V. cholerae infections? | 80.4% | 43 | 65% |
| Q123 | Delivery | WASH | What are the factors / determinants that lead to sustainable investments in water, sanitation, and hygiene at country level? | 80.3% | 44 | 66% |
| Q36 | Description | CM | What are the common cholera treatment complications in vulnerable populations (for example: pregnant women, the elderly, those with severe acute malnutrition)? | 80.2% | 45 | 66% |
| Q107 | Development | Cross-cutting  (All Pillars) | What is the relative and combined impact of different approaches (e.g. case-area targeted interventions (CATI), cluster-based approach, hospital based interventions, etc.) on cholera transmission and mortality at different stages of a cholera outbreak or in different contexts (e.g. during floods, in insecure areas, or hard-to-reach populations)? | 80.1% | 46 | 63% |
| Q42 | Development | CM | Would ReSoMal formulated with higher sodium, or standard oral rehydration solution containing high potassium, result in lower mortality or morbidity, compared to the standard WHO rehydration solution, in children with severe acute malnutrition? | 80.1% | 47 | 66% |
| Q35 | Development | CM | What are valid and reliable hydration status assessment criteria in vulnerable patients with cholera (for example: pregnant women, the elderly, those with acute severe malnutrition)? | 79.8% | 48 | 65% |
| Q85 | Discovery | OCV | Research and development of new or improved vaccines to contribute to accelerate the achievement of the Cholera Roadmap goals. | 79.5% | 49 | 65% |
| INT1 | Development | CM | What is the role of nutritional support during a cholera outbreak, particularly for children? | 79.5% | 50 | 63% |
| Q143 | Development | WASH | What is the most effective practices and technology to collect, manage, and dispose cholera effluent, including identifying alternative technologies? | 79.3% | 51 | 67% |
| Int15 | Delivery | Cross-cutting  (All Pillars) | What are innovative models for promoting multi-sectoral collaboration for cholera prevention and control? | 79.2% | 52 | 62% |
| Q45 | Description | Epi / Sur / Lab | What are the roles of short-term and long-term environmental reservoirs in the transmission of | 78.6% | 53 | 63% |
| Q145 | Development | WASH | What is the impact and cost effectiveness on disease transmission of different components of the water, sanitation, and hygiene package, considering different contexts? | 78.4% | 54 | 63% |
| Q46 | Description | Epi / Sur / Lab | To what extent do asymptomatic infections contribute to transmission of cholera and to long-term latency of cholera in the community? | 78.4% | 55 | 62% |
| Int132 | Delivery | Epi / Sur / Lab | How can mobile alerts and social media platforms be used for detection and surveillance of cholera? | 78.3% | 56 | 60% |
| Q37 | Description | CM | What are the underlying co-morbidities that increase the risk of poor cholera outcomes? | 78.3% | 57 | 63% |
| Int8 | Development | Epi / Sur / Lab | Will community-based reporting by lay persons improve surveillance for cholera (where and how can it be used to optimise surveillance)? | 77.9% | 58 | 64% |
| Q19 | Development | CM | Could zinc or other agents (e.g., boiled green bananas) play a role in reducing cholera severity and deaths in adults? | 77.3% | 59 | 61% |
| Int58 | Delivery | Cross-cutting  (All Pillars) | What is the economic impact of cholera (including outbreaks) and the return on investments and budgetary impact of the various cholera interventions? | 77.2% | 60 | 62% |
| Q6 | Delivery | Cross-cutting  (CM; WASH) | Are there synergistic effects on cholera burden, transmission and mortality when antibiotic prophylaxis is combined with WASH? | 77.0% | 61 | 62% |
| Q9 | Development | CM | What is the effectiveness of antibiotic treatment in vulnerable populations (for example: pregnant women, the elderly, those with severe acute malnutrition) with cholera and with no or some dehydration? | 76.5% | 62 | 61% |
| Int177 | Delivery | Cross-cutting  (CM; CE) | How can we sensitize/motivate men to adopt preventive strategies and seek appropriate care for cholera? | 76.1% | 63 | 60% |
| Q44 | Description | Epi / Sur / Lab | How does individual / community level transmission dynamics of cholera compare with macro-scale (e.g., national / regional level) transmission dynamics in informing control strategies? | 76.0% | 64 | 57% |
| Int61 | Description | Epi / Sur / Lab | What is the optimal number/proportion of cases that need to be laboratory confirmed during the different phases of a cholera outbreak? | 75.9% | 65 | 60% |
| Q17 | Development | CM | What is the sensitivity and specificity of a composite clinical score for diagnosis of cholera in the community compared to cholera confirmed by rapid diagnostic test? | 75.9% | 66 | 60% |
| Q38 | Development | CM | What are the benefits and risks of antibiotic prophylaxis in vulnerable populations in a cholera outbreak (for example: pregnant women, the elderly, those with severe acute malnutrition)? | 75.8% | 67 | 61% |
| Exp3 | Description | Epi / Sur / Lab | What impact does implementing cholera control and prevention interventions in one setting or region have on long-range transmission routes and incidence of cholera in other areas (e.g. South Asia and Africa)? | 75.7% | 68 | 58% |
| Q48 | Description | Epi / Sur / Lab | What are the extrinsic factors that influence the reproduction number (R0) of cholera (e.g. rainfall, crowding etc.)? | 75.7% | 69 | 60% |
| Q73 | Delivery | Cross-cutting (OCV; CE) | Do breast-feeding mothers who receive oral cholera vaccine transfer any level of protection, such as maternal antibodies, against cholera to their infants? | 75.6% | 70 | 59% |
| Q2 | Development | CM | What effect does treatment with antibiotics versus no antibiotics have on mild cholera disease (e.g., diarrhoea with no signs of dehydration)? | 75.5% | 71 | 62% |
| Q34 | Delivery | Cross-cutting  (All Pillars) | What is the effectiveness of different behaviour change theories and methods used in cholera prevention and control programmes? | 75.1% | 72 | 57% |
| Q4 | Development | CM | What is the optimal treatment schedule for mass antibiotic prophylaxis in high-risk gatherings (for example: refugee camps and prisons) and does this have an effect on the magnitude, transmission and secondary attack rate of cholera outbreaks? | 75.0% | 73 | 61% |
| Q124 | Delivery | WASH | What is the role and effectiveness of applying public health policies and regulations (e.g. regarding food safety, open defecation and ban on surface water consumption) in controlling cholera outbreaks? | 75.0% | 74 | 59% |
| Q84 | Delivery | OCV | Under what conditions would the creation of national stockpiles of oral cholera vaccine result in a cost-effective approach to cholera control? | 75.0% | 75 | 57% |
| Q11 | Description | Epi / Sur / Lab | What should be the specific laboratory criteria and cut-off values (Minimum Inhibitory Concentration) for antibiotic susceptibility testing in V. cholerae? | 74.5% | 76 | 58% |
| Q58 | Description | Epi / Sur / Lab | Can real time modelling be used to conduct programme planning and optimise cholera outbreak response? | 74.5% | 77 | 57% |
| Q149 | Description | Epi / Sur / Lab | What are the phenotypic and genotypic characteristics of antibiotic resistance and what are the mechanisms of resistance in V. cholerae? | 73.7% | 78 | 57% |
| Q146 | Description | CM | Does antibiotic resistance have an effect on severity of cholera? | 73.7% | 79 | 58% |
| Q144 | Delivery | WASH | What is the relative impact of water, sanitation, and hygiene investment on national and household budgets? Are the costs well-accepted? | 73.4% | 80 | 56% |
| Q122 | Development | Cross-cutting  (All Pillars) | What are the indicators to measure the impact of behaviour change? | 73.3% | 81 | 55% |
| Q7 | Development | CM | To what extent would large-scale use of antibiotic prophylaxis (either to prevent or respond to an outbreak) affect antimicrobial resistance (AMR) in V. cholerae and/or other pathogens (such as E coli & S pneumoniae)? | 72.9% | 82 | 56% |
| Int145 | Discovery | Epi / Sur / Lab | Research to contribute to the collection of genomic data to create a global V. cholerae sequences database to map long-range transmission routes. | 72.8% | 83 | 56% |
| Q47 | Description | Epi / Sur / Lab | What is the bacterial load of V. cholerae in different sample types/sources, including fresh stools, and how does this correlate with infectious dose, transmission, and disease severity? | 72.7% | 84 | 58% |
| Q134 | Development | CM | Does zinc prophylaxis prevent cholera when given to the contacts of a cholera index case? | 71.9% | 85 | 55% |
| Q150 | Description | Epi / Sur / Lab | Can the epidemic potential of V. cholerae strains be predicted based on their genetic lineage and/or genomic data? | 71.4% | 86 | 54% |
| Q92 | Delivery | Cross-cutting (WASH; CE) | What are the optimal strategies, including behaviour change strategies, for delivering interventions related to safe burial practices and funeral hygiene? | 71.0% | 87 | 54% |
| Q56 | Development | Epi / Sur / Lab | In what settings should serological surveillance be used to monitor cholera transmission? What would be the optimal study design and assays to use for these studies? | 69.6% | 88 | 54% |
| Q12 | Development | CM | What proportion of patients have already received antibiotics before presenting to a cholera treatment centre and does this have an effect on outcomes? | 69.3% | 89 | 55% |
| Int155 | Delivery | Cross-cutting  (All Pillars) | Is there stigma attached at individual level to cholera and the use of cholera interventions and does the stigma influence the disease reporting or uptake of interventions? | 69.3% | 90 | 54% |
| Q90 | Development | WASH | What is the cost-effectiveness of food safety and food hygiene intervention for cholera prevention? | 69.0% | 91 | 51% |
| Q40 | Description | CM | What is the risk of foetal loss during pregnancy in cholera patients? | 65.7% | 92 | 53% |
| Q139 | Description | Epi / Sur / Lab | Does immunity have a role in the emergence of new strains of V. cholerae and what are the best immune markers and methods to explore this hypothesis? | 65.7% | 93 | 50% |
