# Supplementary material for: Application of the Child Health and Nutrition Research Initiative (CHNRI) methodology to prioritize research to enable the implementation of Ending Cholera: A global roadmap to 2030
Source: PLoS One. 2022 May 26;17(5):e0264952. doi: 10.1371/journal.pone.0264952 (PMC9135262; doi:10.1371/journal.pone.0264952)
Supplement: S1 File — (DOCX) [file pone.0264952.s002.docx]

# S1. Interview and survey questionnaire used to adapt CHRNI approach and identify research questions

## Interview questions

1. Please provide your organization name, your primary area of expertise (following Cholera Roadmap pillars: Epidemiology, surveillance, Case Management, OCV, WASH, or Community Engagement), and primary job function (Researcher, implementor, policy / decision maker, donor)
2. In your opinion, in order to achieve the cholera roadmap goals, should research focus more on scaling up the use of existing interventions or on developing novel interventions?

The next set of questions focuses on obtaining your feedback to identify and select criteria that will be used to evaluate and prioritise the cholera research questions. The criteria and their statements will be used as part of the prioritisation process where the respondents will be asked to score whether each research question meets the criteria. Please refer to Table 1, which provides the criteria and their descriptions.

Table 1: Potential prioritization criteria

| **Criterion** | **Description** |
| --- | --- |
| Relevancy | Will the proposed research contribute to addressing relevant evidence gaps in the context of the Cholera Roadmap? |
| Equity | Would you say that the proposed research has the potential to address the existing inequities in cholera disease burden by 2030? |
| Ethical and Answerability | Do you think the proposed research is ethically answerable in the population of interest? |
| Fundability | Do you think that the proposed research is likely to receive funding support? |
| Impact | What would be the contribution of the research outputs to achieving the goal of the cholera roadmap of reducing cholera deaths by 90%? |
| Implementability | Will the proposed research lead to solutions that are:   - - Implementable (introduction and scale up) in the populations of interest?   - Affordable to the countries where they are needed?   - Sustainable over time without external financial or technical support? |

1. Relevancy: should this criterion be used to prioritize proposed research topics? Does the statement capture the criterion? If not, how would you modify it? How important is the criterion to assess proposed research, please choose one option: Extremely important, Very important, Moderately important, Slightly important, Not important at all
2. Equity: should this criterion be used to prioritize proposed research topics? Does the statement capture the criterion? If not, how would you modify it? How important is the criterion to assess proposed research, please choose one option: Extremely important, Very important, Moderately important, Slightly important, Not important at all
3. Ethical and Answerability: should this criterion be used to prioritize proposed research topics? Does the statement capture the criterion? If not, how would you modify it? How important is the criterion to assess proposed research, please choose one option: Extremely important, Very important, Moderately important, Slightly important, Not important at all
4. Fundability: should this criterion be used to prioritize proposed research topics? Does the statement capture the criterion? If not, how would you modify it? How important is the criterion to assess proposed research, please choose one option: Extremely important, Very important, Moderately important, Slightly important, Not important at all
5. Impact: should this criterion be used to prioritize proposed research topics? Does the statement capture the criterion? If not, how would you modify it? How important is the criterion to assess proposed research, please choose one option: Extremely important, Very important, Moderately important, Slightly important, Not important at all
6. Implementability (implementable): should this criterion be used to prioritize proposed research topics? Does the statement capture the criterion? If not, how would you modify it? How important is the criterion to assess proposed research, please choose one option: Extremely important, Very important, Moderately important, Slightly important, Not important at all
7. Implementability (affordable): should this criterion be used to prioritize proposed research topics? Does the statement capture the criterion? If not, how would you modify it? How important is the criterion to assess proposed research, please choose one option: Extremely important, Very important, Moderately important, Slightly important, Not important at all
8. Implementability (sustainable): should this criterion be used to prioritize proposed research topics? Does the statement capture the criterion? If not, how would you modify it? How important is the criterion to assess proposed research, please choose one option: Extremely important, Very important, Moderately important, Slightly important, Not important at all
9. Identify up to three key problem statements / areas and their associated public health impact that may require additional research
10. Roadmap strategy is currently based on pillars: (i) Detect / Describe: Surveillance / epidemiology; (ii) Treat: case management; (iii) Prevent / Protect: OCV, WASH, and community engagement
11. Please provide any additional comments / feedback

## Survey questions

1. Are you currently based in or work in a cholera affected country?
2. Please indicate the country that you are based in.
3. What type of organisation are you affiliated with?
4. Based on the GTFCC Working Groups, please indicate your primary area of expertise.
5. Are you currently engaging with the GTFCC (e.g., a member, contributing to current cholera discussions, and / or attending working group meetings)
6. Please provide a high level overview of your job function and ongoing work. Please highlight any activities related to cholera.
7. Indicate your primary job function.
8. Is it more important to you that the research activities, including operational, implementation, and behavioural research, are used to (i) scale-up or improve coverage of existing interventions; (ii) develop new interventions/knowledge; and (iii) other, describe. On a scale from 1 to 5, please choose Extremely Important or Not important at all.

You have selected that you are affiliated with a donor. We would like to gather high level feedback regarding the donor perspective on the Cholera Roadmap Research Agenda. This information will help to inform the defined context for the Cholera Roadmap Research Agenda.

1. Please provide an estimate of the average sized grant that your institution provides to fund research activities.
2. Describe your preferred style of investment.
3. Please add any other considerations from a donor perspective that are important for the development of the Research Agenda.

The next set of questions focuses on obtaining your feedback to identify and select criteria that will be used to evaluate and prioritise the cholera research questions. The criteria and their statements will be used as part of the prioritisation process where the respondents will be asked to score whether each research question meets the criteria.

1. How important do you feel the following questions are to evaluate each of the research questions? (Extremely important to Not at all important)

- EQUITY: Would you say that the proposed research has the potential to address the existing inequities in cholera disease burden by 2030?
- ETHICAL AND ANSWERABILITY: Do you think the proposed research is ethically answerable in the population of interest
- FUNDABILITY: Do you think that the proposed research is likely to receive funding support?
- IMPACT: What would be the impact of the proposed research on achieving the goals and objectives of the Cholera Roadmap 2030?
- IMPLEMENTABILITY: Will the proposed research lead to solutions that are implementable (introduction and scale up) in the populations of interest?
- IMPLEMENTABILITY: Will the proposed research lead to solutions that are affordable to the countries where they are needed?
- IMPLEMENTABILITY: Will the proposed research lead to solutions that are sustainable over time without external financial or technical support?
- RELEVANCY: Will the proposed research contribute to address relevant evidence gaps in the context of the Cholera Roadmap?

1. Please provide any additional comments regarding the prioritisation criteria (e.g., if there are any missing criteria, rephrasing criteria, etc.)

The next set of questions are designed to collect your input on key problems and associated knowledge gaps related to the Cholera Roadmap, we are also requesting that you provide a brief description of the public health impact if the problem was addressed. Please note you will only be allowed to submit up to three (3). The information you provide will be cross-checked against an existing list and added if a gap is identified.

1. Would you like to submit problem statements and their associated knowledge gaps and public health impact? Note you may submit up to three (3).
2. Provide below a description of the problem statement, knowledge gaps, and, its anticipated health impact. You may optionally provide a proposed research question and other information (e.g., implications on other Roadmap pillars). You may provide up to 3 potential problem statements.
