# Supplementary material for: Application of the Child Health and Nutrition Research Initiative (CHNRI) methodology to prioritize research to enable the implementation of Ending Cholera: A global roadmap to 2030
Source: PLoS One. 2022 May 26;17(5):e0264952. doi: 10.1371/journal.pone.0264952 (PMC9135262; doi:10.1371/journal.pone.0264952)
Supplement: S2 File — (DOCX) [file pone.0264952.s003.docx]

# S2. Phase 1 results

Four hundred and fifty-three research questions were identified. The identified research questions were reviewed for clarity and duplication and discussed bi-laterally with key experts and GTFCC working group chairs. Removal of duplicates questions, those that had been adequately addressed in previous research and merging related questions resulted in 124 research questions. The 124 research questions underwent a final review by 17 experts representing all of the Roadmap pillars, which resulted in the finalization of 93 research questions. Both WASH and community engagement had a significant proportion of their research questions classified as in the category of more than one pillar (seven of 17 WASH-related questions and all tencommunity-engagement questions were classified into this category). Further, there were an additional 12 research questions that were identified as cutting across all of the Roadmap pillars.

The figure below shows the break-out of the final research questions by Roadmap pillar and 4D framework. Annex 1 provides the comprehensive list of research questions.

**Figure 1: Final research questions by Roadmap pillar and 4D framework**

Table 2 provides the demographics of the individuals who responded.

**Table 2: Demographics of 141 individuals consulted as part of Phase one**

| **Demographics** | **Interviews** | **Survey** | **Total** | **%** |
| --- | --- | --- | --- | --- |
| **Expertise** |  |  |  |  |
| Epidemiology / Surveillance / Laboratory | 30 | 45 | 75 | 35% |
| OCV | 12 | 34 | 46 | 22% |
| WASH | 9 | 27 | 36 | 17% |
| Case Management | 9 | 12 | 21 | 10% |
| Community Engagement | 9 | 16 | 25 | 12% |
| Other | 9 | 0 | 9 | 4% |
| **Organization Type** |  |  |  |  |
| Impl. partner (CDC, Int'l Org, UN, CSO, and NGO) | 20 | 35 | 55 | 39% |
| Academic / Research | 25 | 32 | 57 | 40% |
| Donor | 0 | 14 | 14 | 10% |
| Gov't in cholera endemic countries | 9 | 4 | 13 | 9% |
| Independent | 0 | 2 | 2 | 1% |
| **Job function** |  |  |  |  |
| Researcher | 26 | 39 | 65 | 37% |
| Implementing partner | 15 | 38 | 53 | 30% |
| Donor | 0 | 14 | 14 | 8% |
| Implementer | 15 | 7 | 22 | 13% |
| Policy / decision maker | 14 | 6 | 20 | 11% |
| **Respondent location** |  |  |  |  |
| Global, includes EUR and AMR regions, excluding Haiti | 14 | 62 | 76 | 54% |
| AFR | 22 | 12 | 34 | 24% |
| SEAR | 13 | 9 | 22 | 16% |
| EMR | 2 | 3 | 5 | 4% |
| Haiti | 1 | 1 | 2 | 1% |
| WPR | 2 | 0 | 2 | 1% |
|  |  |  |  |  |
| **Total** | **54** | **87** | **141** |  |

Although rating the importance of the criteria differed between the interviews and surveys, there was a convergence of opinions on the top four criteria of Relevancy, Impact, Implementability, and Ethical Answerability. Fundability (of research projects) was consistently rated as the least important criterion in both interviews and surveys.

The interviewees had a larger spread on the level of importance across the different criteria compared to the respondents via the online survey. This could be due to the limitations of the online survey that did not allow any discussion or explanation of the criteria.

The stratified analyses did not reveal major differences in the rating of the potential criteria; however the results for considering those at global levels had a wider spread on the perceived level of importance of the potential criteria compared to those located in cholera-affected countries.

For the open-ended responses, the respondents had minor refinements to the definitions of impact, implementability, relevancy, and sustainability criteria, these were generally accepted as relevant criteria to prioritize research questions. In comparison, many felt that that the fundability and affordability would limit the potential research activities and not allow for sufficient levels of innovation (e.g., research outputs can improve affordability). Finally, the respondents agreed that equity and ethical were important considerations, but indicated that these criteria would not differentiate between potential research activities given that cholera is inequitably distributed and there is insufficient information to determine whether the research activities follows ethics protocols. The feedback resulted in the selection and finalization of the five criteria.

The Research Agenda Steering Committee in consultation with 48 key stakeholders, including donors, implementing partners, and experts, agreed to the following contextual factors for the Research Agenda:

- **Population of interest**: All countries and communities where cholera is endemic and/or there is epidemic risk of cholera
- **Time scale**: Present day to 2030
- **Geographic scope of research**: Global, regional, national, and sub-national levels. Sub-national may include different administrative levels, such as provinces or states, districts, communities or households
- **Impact of interest**: Reduction of deaths and burden of cholera. Burden may include prevalence and morbidity as well as any economic or social impact of cholera

Finally, 40 individuals responded to the online survey to distribute 100 points across the key criteria, approximately 50% represented global or headquarter perspectives and the remaining 50% represented regional or country perspectives. The following weights were identified:

- Impact: 1·20
- Implementability: 1·12
- Relevancy: 1·06
- Sustainability: 0·83
- Answerability: 0·79

# 
