# Supplementary material for: Application of the Child Health and Nutrition Research Initiative (CHNRI) methodology to prioritize research to enable the implementation of Ending Cholera: A global roadmap to 2030
Source: PLoS One. 2022 May 26;17(5):e0264952. doi: 10.1371/journal.pone.0264952 (PMC9135262; doi:10.1371/journal.pone.0264952)
Supplement: S3 File — (DOCX) [file pone.0264952.s004.docx]

# S3. Guidance document accompanying prioritization survey to evaluate research questions

# Purpose

This document aims to provide additional guidance to assist the cholera community for applying the prioritisation criteria to the research questions.

# Background

To develop the prioritised Cholera Roadmap Research Agenda, the Child Health and Nutrition Research Initiative (CHNRI) approach is being applied. The CHNRI approach utilizes a crowd sourcing technique that relies on the opinion, knowledge, and experiences of various stakeholders to evaluate the importance of the research questions. Your input, together with those of other stakeholders, will be compiled to develop a “priority score” for each research question. Please note that all outcomes of this exercise will be documented in the final Cholera Roadmap Research Agenda.

## Options to evaluate the research questions

You will be asked to apply each criterion (see Table 1 for short descriptions of the criteria) to each of the research questions, taking the defined context into consideration. In doing so, you will have the following four options for responding:

- **Yes**: In your opinion, the research question meets the particular criterion.
- **No**: In your opinion, the research question does not meet the particular criterion.
- **Maybe**: In your opinion, it is uncertain that the research question meets the particular criterion.
- **I don’t know**: You feel you do not have sufficient knowledge or experience to evaluate the specific research question.

The four potential responses will be assigned the following values to calculate the prioritisation score for each research question.

- Yes = 1
- No = 0
- Maybe = 0.5
- Don’t know = response will not be included in the prioritisation analysis

Please answer "Yes", "No", or "Maybe" to as many questions as you feel able to, they do not necessarily need to be within your area(s) of interests or expertise. Please only select "I don’t know" if you really feel that you do not have sufficient knowledge or experience to evaluate the specific research question. In order to achieve greater uniformity when scoring the research questions, the following description of the defined Cholera Roadmap Research Agenda context is provided to be taken into consideration when applying each criterion to the research questions.

Application of the prioritisation criteria using the defined context.

## Context

The context determines the scope of the research within the Cholera Roadmap Research Agenda focusing on the who, when, where and what outcomes are needed from the research activities. As the research questions will be interpreted based on your individual perspective and experience, the context aims to ensure that “everyone is on the same page” when scoring the research questions. The following four contextual factors have been defined:

- Population of interest: All countries and communities where cholera is endemic and/or there is epidemic risk of cholera
- Time scale: Present day to 2030
- Geographic scope of research: Global, regional, national, and sub-national levels. Sub-national may include different administrative levels, such as provinces or states, districts, communities or households
- Impact of interest: Reduction of deaths and burden of cholera. Burden may include prevalence and morbidity as well as any economic or social impact of cholera

## Prioritisation criteria

Table 2 provides short descriptions of the prioritisation criteria with a more detailed explanation of each criterion in the text.

**Table 2: Short descriptions of prioritisation criteria**

| **Criterion** | **Short Description** |
| --- | --- |
| Answerability | Do you think the proposed research is answerable in cholera-affected countries and communities?  *Assumes all protocols will be subject to appropriate ethics reviews. |
| Impact | Will the research outputs contribute to reducing cholera deaths and burden? |
| Implementability | Will the proposed research lead to solutions that are implementable (e.g. feasibility of introduction, including acceptability to the cholera-affected communities and scale up)? |
| Relevancy | Will the proposed research contribute to addressing relevant evidence gaps in the cholera-affected countries or communities when implementing the Cholera Roadmap? |
| Sustainability | Will the proposed research lead to solutions that are sustainable over time without, or with only limited, external financial or technical support in cholera-affected countries? |

## Answerability

Based on your opinion and experience, do you believe that plans to answer the research question can be designed and conducted^*^ considering the following contextual factors:

- Can the research activities be designed and conducted in the population of interest (e.g., countries and communities where cholera is endemic or there is epidemic risk of cholera)?
- Will the research question generate results by the defined time scale?

***** assuming that all research protocols would be subject to and follow established ethical processes and reviews

## Impact

Based on your opinion and experience, do you believe the proposed research question will contribute to the defined impact of reducing cholera deaths and burden (includes prevalence, morbidity as well as economic or social impact of disease). As the research questions cover a wide breadth of research types, it is important to consider how the various types of research can contribute to impact either directly or indirectly. For example, will the outputs (e.g., **Research output** can refer to anything that is generated via the research activities such as the new knowledge regarding cholera, new interventions to address cholera, or improvements to the delivery of existing interventions.) of the research contribute directly or indirectly to reduce cholera burden by:

- improving the understanding of cholera; or
- improving the delivery and uptake of existing interventions or optimizing their effectiveness; or
- developing novel interventions or delivery strategies.

## Implementability

Based on your opinion and experience, do you believe that the outputs generated from the research activities will lead to solutions that would be feasible to introduce and be accepted by the population of interest (e.g., countries and communities where cholera is endemic or where there is epidemic risk of cholera)?

Similar to Impact criterion, given the wide breadth of research questions it is important to consider all aspects of how research leads to implementable solutions e.g., research answering questions which generate knowledge or improve understanding of the disease itself and people’s attitudes to it can contribute to more effective implementation of interventions.

## Relevancy

Based on your opinion and experience, do you believe that this research question contributes to filling key evidence gaps that are hindering the achievement of the Roadmap goals and will the outputs of the research be applicable to cholera-affected countries and communities?

## Sustainability

# Based on your opinion and experience, do you believe that the outcomes or solutions of the research question will continue to be used by cholera-affected communities over the medium and long-term, including beyond 2030 without, or with only, external financial or technical assistance?
